# Large Area Emission in p-Type Polymer-Based Light-Emitting Field-Effect Transistors by Incorporating Charge Injection Interlayers

**DOI:** 10.3390/ma14040901

**Published:** 2021-02-14

**Authors:** Gizem Acar, Muhammad Javaid Iqbal, Mujeeb Ullah Chaudhry

**Affiliations:** 1 Faculty of Engineering and Natural Sciences, Sabanci University, Istanbul 34956, Turkey; gizem.acar@durham.ac.uk; 2 Department of Engineering, Durham University, South Rd, Durham DH13LE, UK; 3 Centre of Excellence in Solid State Physics, University of the Punjab, Lahore 54590, Pakistan; javaid.cssp@pu.edu.pk

**Keywords:** organic light emitting field effect transistors (LEFETs), p-type LEFETs, organic electronics, polymers, display pixilation

## Abstract

Organic light-emitting field-effect transistors (LEFETs) provide the possibility of simplifying the display pixilation design as they integrate the drive-transistor and the light emission in a single architecture. However, in p-type LEFETs, simultaneously achieving higher external quantum efficiency (EQE) at higher brightness, larger and stable emission area, and high switching speed are the limiting factors for to realise their applications. Herein, we present a p-type polymer heterostructure-based LEFET architecture with electron and hole injection interlayers to improve the charge injection into the light-emitting layer, which leads to better recombination. This device structure provides access to hole mobility of ~2.1 cm^2^ V^−1^ s^−1^ and EQE of 1.6% at a luminance of 2600 cd m^−2^. Most importantly, we observed a large area emission under the entire drain electrode, which was spatially stable (emission area is not dependent on the gate voltage and current density). These results show an important advancement in polymer-based LEFET technology toward realizing new digital display applications.

## 1. Introduction

The possibility of solution-processed active-matrix organic light-emitting diode (AMOLED)-based displays has attracted significant interest from the scientific and industrial communities due to their scope of producing low cost and high throughput displays [1,2,3]. However, the back-plane poly-Si transistors, which drive the individual AMOLED pixels, limits the scope of fully solution-processed displays [4,5]. Light-emitting field-effect transistors (LEFETs) are emerging technology and offer an alternative route to fully solution-processed pixels [6,7,8,9]. LEFETs provide a combination of the switching function with the light emission, which enables a promising new structure for studying optoelectronics and developing new display applications [4,6,7,8,9,10,11,12,13,14,15,16,17,18,19,20].

Since the first report in 2003 [19], LEFETs have brought a different perspective to organic semiconductors, paving the way for research in device architecture and material development to simultaneously study charge carrier transport and light emission [13,16,17,18]. Multiple device geometries and material combinations have been suggested to improve the LEFET parameters such as external quantum efficiency (EQE) [21,22,23,24], charge carrier mobility [25,26,27,28], aperture ratio [29,30], current ON/OFF ratio [20,31], and large area emission [32,33]. However, LEFETs are far from realizing into commercial products due to their limited performance. A multilayer LEFET can provide higher mobility and lead to better charge injection in LEFETs, but their EQE at higher brightness is somewhat limited. A semitransparent electrode enables the higher EQE in multilayer LEFETs by outcoupling the trapped light underneath it; however, it does not provide a uniform and stable emission area, which is essential for pixilation [21,34]. More recently, n-type hybrid LEFETs (a combination of the inorganic charge transport layer and organic emissive layer) have been reported to show a large, uniform, and stable light-emitting area through interface doping [7,35,36]. However, for all organic p-type LEFETs, this has not been achieved.

In this paper, we report a device architecture of a p-type polymer-based LEFET that simultaneously provides higher charge carrier mobility, higher EQE, and uniform and stable emission. The device structure consists of: (i) polymer charge transport layer of poly[4-(4,4-dihexadecyl-4H-cyclopenta[1,2-b:5,4-b′]-dithiophen-2-yl)-*alt*-[1,2,5]thiadiazolo-[3,4-*c*]pyridine] (PCDTPT) leading to hole mobility of 2.1 cm^2^ V^−1^ s^−1^; (ii) an emissive layer of PPV based polymer PDY-132 (also known as Super Yellow) provides an EQE of 1.6% at 2600 cd/m^2^; (iii) a Mo_2_O_3_ layer under source electrode enabling better injection of the holes; and (iv) a Mo_2_O_3_ interfacial layer between both polymers, enabling a large area emission under the drain electrode. This new device architecture opens new doors to study the interfacial doping in polymers and new polymer-based display applications.

## 2. Device Fabrication and Characterization

**Figure 1:**
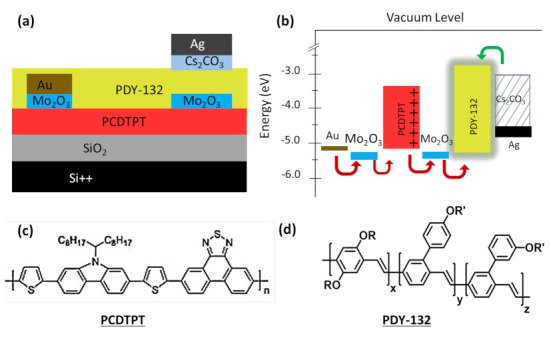
(**a**) Device structure with Mo_2_O_3_ and Cs_2_CO_3_ interlayers and (**b**) energy diagram of active materials and injection layers. Molecular structures of polymers (**c**) PCDTPT and (**d**) PDY–132 (Super Yellow).

The LEFET devices were fabricated using a 300 nm thermally grown layer of SiO_2_ as a dielectric on Si substrates. The substrates were cleaned by ultrasonication in acetone and isopropanol alcohol (IPA) for 15 min each and then blow-dried under pressurized nitrogen. An 80 nm layer of charge transport polymer poly [4-(4,4-dihexadecyl-4H-cyclopenta [1,2-b:5,4b′] dithiophen-2-yl)-alt-[1,2,5] thiadiazolo [3,4-c] pyridine], PCDTPT, was then spin-coated onto substrates from a 5 mg/mL solution in dichlorobenzene. Substrates were then annealed at 150 °C for 30 min. Using an aligned shadow mask-1, a hole injecting asymmetric source contact, consisting of a 10 nm of Mo_2_O_3_ layer and 50 nm of Au layer, was deposited on top of the PCDTPT layer by thermal evaporation in a high vacuum. A 10 nm layer of Mo_2_O_3_ was also deposited using the aligned drain mask-2, to be exactly underneath the drain electrode. A 100 nm layer of polymer PDY–132 was then spin-coated onto the entire substrate from a solution of 10 mg/mL in toluene. The substrates were annealed again at 150 °C for 30 min. Finally, the electron-injecting semitransparent drain electrode, consisting of 10 nm of Cs_2_CO_3_ and 20 nm of Ag layers, was deposited through aligned shadow mask-2 to give a channel length (*L*) and width (*W*) of 100 µm and 2 mm, respectively. The complete device structure is shown in Figure 1a and the energy level diagram with charge injection schematics is shown in Figure 1b, along with the molecular structures of PCDTPT in Figure 1c and PDY–132 in Figure 1d.

Two Agilent B2912A units connected to electrical probes and a calibrated photodetector attached to an EverBeing C-series probe station were used to characterize the LEFETs. A microscope attached to the probe station was used to capture the images. An optical fiber attached to the probe station and an OceanInsight USB Flame spectrometer was used to determine the electro luminance spectrum. A Dektak profilometer was used to measure the film thickness and the photoluminescence quantum yield (PLQY) of thin films was measured using the de Mello method [37] as reported previously. The brightness of the LEFET was measured by comparing the photocurrent in a photodetector with that of a reference device of known light emission area and brightness. The correct brightness value was measured by correcting the photocurrent for the effective light-emission area. The brightness of the reference device was measured with the help of a Minolta Candela meter (LS-100). The values of brightness and EQE were found using the standard procedure described elsewhere [20,36,38,39,40,41].

## 3. Results and Discussion

**Figure 2:**
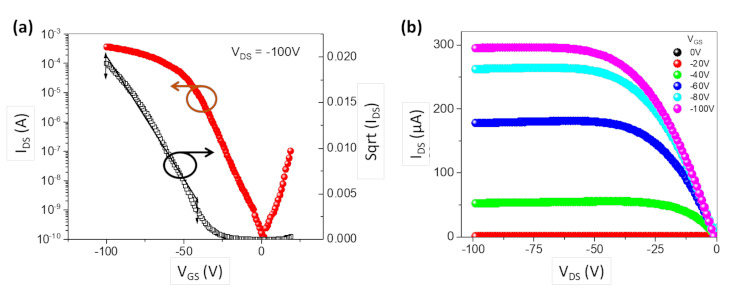
(**a**) Transfer characteristics of the light-emitting field-effect transistor (LEFET) (red) and the square root of the source-drain current (black) with a linear fit for channel length L = 100 µm, and channel width = 2400 µm. (**b**) Output characteristics of the p-type LEFET at various gate voltages.

Figure 2a,b shows the transfer and output characteristic curves of the LEFET, respectively. A current ON/OFF of >10^6^ was achieved in the saturation regime. The charge carrier mobility (*µ*) in the saturation regime was calculated from the transfer characteristics given in Figure 2a using Equation (1).

(1) $$I_{DS}=\frac{W\;C_{i}}{2\;L}\;\mu \;{\left(V_{GS}-V_{TH}\right)}^{2}$$

where *I_DS_* is the measured source-drain current; *W* and *L* are the width and length of the device channel, respectively; *V_GS_* is the corresponding applied gate voltage; and *C_i_* is the capacitance of the SiO_2_ dielectric. A linear fit was applied to the sqrt (*I_DS_*) in the extensive range of the curve to provide the average hole mobility and limit the error in estimating the mobility. The threshold voltage (*V_TH_*) was extracted using the linear extrapolation of sqrt (*I_DS_*) of the transfer curve in Figure 2a. The charge carrier mobility was estimated to be 2.1 cm^2^ V^−1^ s^−1^, reflecting the mobilities associated with the PCDTPT polymer without the PDY-132 layer [42] as a result of non-planar contact geometry [11], which eliminates the injection barrier at the source electrode.

**Figure 3:**
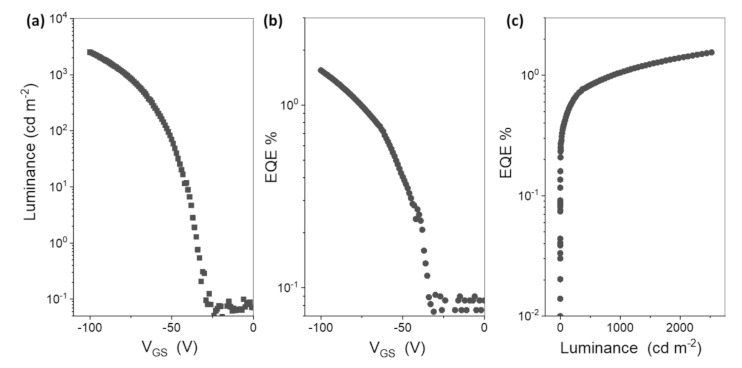
(**a**) Optical transfer characteristic of the LEFET showing luminance versus gate voltage, (**b**) external quantum efficiency (EQE) versus gate voltage and (**c**) EQE versus Luminance.

Figure 3a shows the optical transfer characteristics of luminance vs. gate voltage of the LEFET device. Bright yellow light emission of the peak luminance of 2600 cd m^−2^ was observed under the drain electrode. An EQE of 1.6%, as shown in Figure 3b, was estimated at a high luminance of 2600 cd m^−2^. The EQE vs. luminance graphs in Figure 3c showed an increase in EQE trend, which is an exciting feature and needs a follow-through in LEFET devices. Usually, at high brightness, the EQE tends to roll-off and decrease with increasing current density. However, the LEFET structure has been reported to lower this roll-off of EQE [34] as observed in these results. The results are summarized in Table 1. The calculated luminous efficiency at maximum current density (or gate voltage of 100 V) was 3.2 lm per Watt.

**Figure 4:**
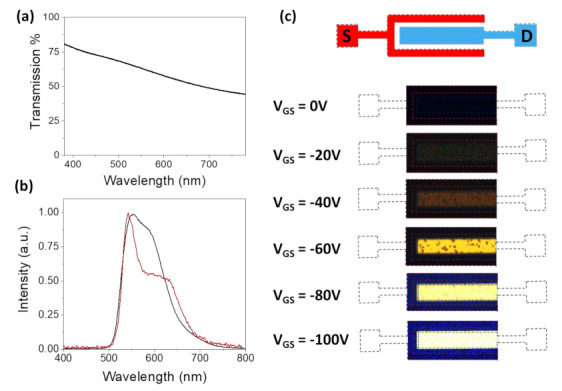
(**a**) Transmission of the electron-injecting electrode (Cs_2_CO_3_/Ag). (**b**) Electroluminescence (red) and photoluminescence (black) spectra of emitted light in LEFET. (**c**) Micrograph of light emission from the drain electrode (0.4 mm × 1 mm) at gate voltages ranging from 0 V to 100 V.

Figure 4a shows the transmission spectrum of the Cs_2_CO_3_/Ag electrode, which was around 60% transparent at 560 nm (the peak emission wavelength of PDY-132). This high transparency of 60% enables the light under the drain electrode to come out through the semitransparent electrode, which could be reflected in the case of an opaque electrode and absorbed by the Si substrate. The photoluminescence (PL) and electroluminescence (EL) spectrum of the emitted light is provided in Figure 4b, which is characteristic of the emissive polymer PDY-132. The EL spectrum was corrected with the reflectance of the Cs_2_CO_3_/Ag electrode. The emission images from the LEFET are shown in Figure 4c. The entire semitransparent Cs_2_CO_3_/Ag electrode emitted light during the operation. The large area emission under the Cs_2_CO_3_/Ag electrode was observed to be spatially stable and its position was independent of the applied gate voltage as shown in the series of images in Figure 4c. Gate voltage was used to modulate the emission intensity. There were some dark spots at a lower voltage, which we attributed to the inhomogeneity of the Mo_2_O_3_ interlayer. However, at higher emission intensity (or gate voltages), the black spots were not visible due to the camera detector’s saturation.

Under bias operation, the holes are injected from the Au/Mo_2_O_3_ (~−5.4 eV) source electrode into the PCDTPT layer (−5.1 eV) and transported along the channel in the PCDTPT layer and injected into HOMO of polymer PDY-132 layer. Incorporating the Mo_2_O_3_ interlayer between the PCDTPT, and PDY-132 further lowered the hole injection barrier from the PCDTPT layer (−5.1 eV) to the HOMO of PDY-132 (−5.3 eV). The electrons from the Cs_2_CO_3_/Ag drain electrode were injected into the LUMO of PDY-132 (−2.9 eV) and recombined to form excitons with injected holes incoming from the p-type channel, as shown in Figure 1b. The light was emitted through the electron injecting semitransparent Cs_2_CO_3_/Ag electrode, as shown in Figure 4c. Previous studies have demonstrated that using a thin layer of Mo_2_O_3_ shifts the work function of semiconductors and metals [11]. The Mo_2_O_3_ layer lowers the injection barrier at the source electrode for hole injection and it also facilitates the hole injection from PCDTPT to PDY-132 at the interface of both polymers. The better injection facilitates the exciton formation and resulting in higher EQE. The efficiency of light emission is given by Equation (2):

(2) $$\varphi_{EQE}=\varphi_{escape}\times \varphi_{capture}\times \varphi_{spin}\times \varphi_{PLQE}$$

where *φ_capture_* (recombination efficiency) is the fraction of electrons-holes that recombine to form excitons; *φ_EQE_* is the calculated EQE; *φ_escape_* indicates the fraction of escaped photons from the device; *φ_spin_* is the spin-statistics factor, and *φ_PLQE_* is the photoluminescence quantum yield (PLQE) in the solid-state. The polymer is a singlet emitter and so *φ_spin_* = 0.25. The *φ_escape_* is approximately 1/2n^2^ (where n is the refractive index) for isotropic emission. The calculated maximum radiative recombination efficiency of the LEFETs was calculated as 48%. We attributed the superior recombination efficiency in this LEFET structure to the better injection due to Mo_2_O_3_ interlayers.

## 4. Conclusions

In summary, we have demonstrated a polymer-based p-type LEFET in a heterostructure device architecture with hole mobility approaching 2.1 cm^2^ V^−1^ s^−1^ and a current ON/OFF >10^6^. The incorporation of the Mo_2_O_3_ interlayer between the polymer leads to superior operating characteristics including an EQE of 1.6% at a luminance of 2600 cd m^−2^ and, more importantly, a large area, uniform, and stable light emission under the entire drain electrode. The results here demonstrate the feasibility of the solution-processable p-type polymer-based LEFET technology for applications in the future-generation of high-definition displays.

## Figures and Tables

**Table 1:** Key results of the light-emitting field-effect transistor (LEFET).

Hole Mobility	Current ON/OFF	EQE	Luminance
2.1 cm^2^ V^−1^ s^−1^	>10^6^	1.6%	2600 cd m^−2^

## Data Availability

All data included in this study are available upon request by contact with the corresponding author.
